# Orthology Inference in Nonmodel Organisms Using Transcriptomes and Low-Coverage Genomes: Improving Accuracy and Matrix Occupancy for Phylogenomics

**DOI:** 10.1093/molbev/msu245

**Published:** 2014-08-25

**Authors:** Ya Yang, Stephen A. Smith

**Affiliations:** ^1^Department of Ecology & Evolutionary Biology, University of Michigan, Ann Arbor

**Keywords:** Diplopoda, phylotranscriptomics, RNA-seq, Vitaceae

## Abstract

Orthology inference is central to phylogenomic analyses. Phylogenomic data sets commonly include transcriptomes and low-coverage genomes that are incomplete and contain errors and isoforms. These properties can severely violate the underlying assumptions of orthology inference with existing heuristics. We present a procedure that uses phylogenies for both homology and orthology assignment. The procedure first uses similarity scores to infer putative homologs that are then aligned, constructed into phylogenies, and pruned of spurious branches caused by deep paralogs, misassembly, frameshifts, or recombination. These final homologs are then used to identify orthologs. We explore four alternative tree-based orthology inference approaches, of which two are new. These accommodate gene and genome duplications as well as gene tree discordance. We demonstrate these methods in three published data sets including the grape family, Hymenoptera, and millipedes with divergence times ranging from approximately 100 to over 400 Ma. The procedure significantly increased the completeness and accuracy of the inferred homologs and orthologs. We also found that data sets that are more recently diverged and/or include more high-coverage genomes had more complete sets of orthologs. To explicitly evaluate sources of conflicting phylogenetic signals, we applied serial jackknife analyses of gene regions keeping each locus intact. The methods described here can scale to over 100 taxa. They have been implemented in python with independent scripts for each step, making it easy to modify or incorporate them into existing pipelines. All scripts are available from https://bitbucket.org/yangya/phylogenomic_dataset_construction.

## Introduction

Orthology is a phylogenetic concept as orthologous genes are defined as those genes that have descended from an ancestral sequence of their common ancestor through speciation (Fitch 1970, 2000). Accurate orthology inference is critical for phylogenomic reconstruction and functional studies. However, this inference is especially challenging for data sets using transcriptomes or low-coverage genomes that often contain misassemblies and partial or missing sequences. The complexities of these data types also make it difficult to distinguish recently duplicated copies from allelic variations, splice variants, and misassemblies.

A number of orthology inference methods have been applied to phylogenomic analyses based on transcriptomes and low-coverage genomes, such as orthoMCL (Li et al. 2003), Hcluster_sg (as part of TreeFam; Li et al. 2006), SCaFoS (Roure et al. 2007), HaMStR (Ebersberger et al. 2009), and OrthoSelect (Schreiber et al. 2009). Emerging tools such as OMA-GETHOGs (Roth et al. 2008; Altenhoff et al. 2013) and Agalma (Dunn et al. 2013) have also attracted interest in their phylogenomic applications. Among them, HaMStR is by far the most widely used. HaMStR is based on a modified reciprocal similarity criterion that starts with querying a set of precomputed high-quality orthologs (“core-orthologs”) against candidate sequences (Ebersberger et al. 2009). The resulting significant hits are then queried against all genes in the reference taxon. HaMStR only adds the candidate to the ortholog group if the best hit in the reference taxon is also member of the same ortholog group (Ebersberger et al. 2009). Considering that incomplete sequences, gene and genome duplication, and molecular rate heterogeneity are almost certainly present in most data sets, the reciprocal criterion is frequently violated. A number of other alternative orthology inference pipelines also suffer from using similarity measurements as approximations to directly infer orthology (Li et al. 2003; Roure et al. 2007; Schreiber et al. 2009; Altenhoff et al. 2011, 2013).

Given the incomplete and noisy nature of transcriptomic and low-coverage genomic data, orthology is best inferred by using phylogenies to separate paralogs and orthologs after homology has been established (Gabaldón 2008). A variety of tree-based orthology inference methods have been developed. However, with a few exceptions, most of these tree-based methods require a known species tree. This is often undesirable as many of these data were generated for the purpose of estimating an unknown species tree. PHYLDOG (Boussau et al. 2013) estimates gene trees and the species tree simultaneously taking duplications and gene loss into account. However, it was designed for genomic data. Besides potential scaling issues with such an approach as data sets grow, transcriptomes may lack a particular gene due to silencing or low expression and coverage. Taxa with low gene coverages tend to be grouped together due to shared “gene loss” (Boussau et al. 2013).

An alternative strategy adopted by Agalma (Dunn et al. 2013) and recent implementations of OrthologID (Chiu et al. 2006) consists of two stages: Obtaining homologs and separating orthologs from paralogs. Both pipelines infer homologs using an all-by-all BLAST search (Altschul et al. 1990) followed by Markov clustering (MCL) that identifies sequence clusters based on the relative connectivity (presence/absence of hits) and relative strength of connections (*E* values from BLAST hits) among sequences (van Dongen 2000). A phylogenetic tree is then inferred for each homolog. To obtain orthologs, the two pipelines use different approaches. Agalma takes only the homolog tree topology into account. It looks for the subtree that has the highest number of nonrepeating taxa, cuts it off as an ortholog, and repeats the search and cutting on the remaining tree (Dunn et al. 2013). This approach has the advantage of being relatively assumption free. However, when there are genome duplications, it breaks orthologs into fragments. This is especially problematic when there are multiple, nested genome duplications as is frequently seen in plants (De Smet et al. 2013). On the other hand, orthologID considers both homolog tree topology and the homolog sequence alignment, using a partial guide tree determined from taxa with genome sequences available (Chiu et al. 2006). It is able to accommodate gene and genome duplications, yet it is limited by the availability of annotated genomes required to build the guide tree for each ortholog group. Most areas of the tree of life still lack reasonable whole-genome sequence coverage. Both Agalma and orthologID improve enormously on previous methods by taking gene tree into account (Chiu et al. 2006; Dunn et al. 2013). However, there is the great potential for additional components and methods that would allow for higher flexibility and broader applications.

Here, we outline a flexible orthology inference procedure based on identifying homologs followed by cleaning, aligning, and cutting homolog trees. We demonstrate this approach and compare different methods for cutting homolog trees in three recently published phylogenomic data sets across diverse taxonomic groups and ages. The grape family, or Vitaceae, consists of approximately 900 species with a stem age of approximately 95 Ma (Wen et al. 2013). The grape data set (GRP) (Wen et al. 2013) includes 15 transcriptomes and a proteome from the grape genome annotation. The millipedes (class Diplopoda) are an ancient and diverse group with fossils dating back to 428 Ma (Brewer and Bond 2013). The millipedes data set (MIL) (Brewer and Bond 2013) includes nine transcriptomes, one expressed sequence tag (EST) data set, and two non-millipedes proteomes from genome annotation. The aculeate Hymenoptera includes ants, bees, and wasps and has a crown age of approximately 150 Ma (Wilson et al. 2013). The aculeate Hymenoptera data set (MIL) (Johnson et al. 2013) includes 18 ingroup data sets (11 transcriptomes, 1 low-coverage genome, 6 annotated genomes) and one outgroup from annotated genome.

### New Approaches

Our orthology inference approach is tree-based, does not rely on a known species tree, and is capable of accommodating genome duplications and different outgroup scenarios. It differs from previous published tree-based and species tree-independent orthology inference methods in a number of ways. We take phylogenetic trees into account in both homolog inference as well as ortholog inference. We explore four alternative strategies for obtaining orthologs from cutting homolog trees, two of which are newly proposed to explicitly accommodate gene duplications. To evaluate conflicting phylogenetic signals, we use a jackknife strategy with multiple resampling ratios. This strategy resamples by locus, not by site, and therefore keeps each locus intact and explicitly evaluates conflicting phylogenetic signals among loci. Finally, given the ever-changing landscape of sequence processing, alignment, and tree inference methods, each step of our procedure is written in separate python scripts with a lightweight phylogenetic tree library, which allows for steps to be easily modified, swapped, and moved between computer clusters and desktop machines.

## Results and Discussion

### Homology Inference Using Clusters and Trees

**Fig. 1.**
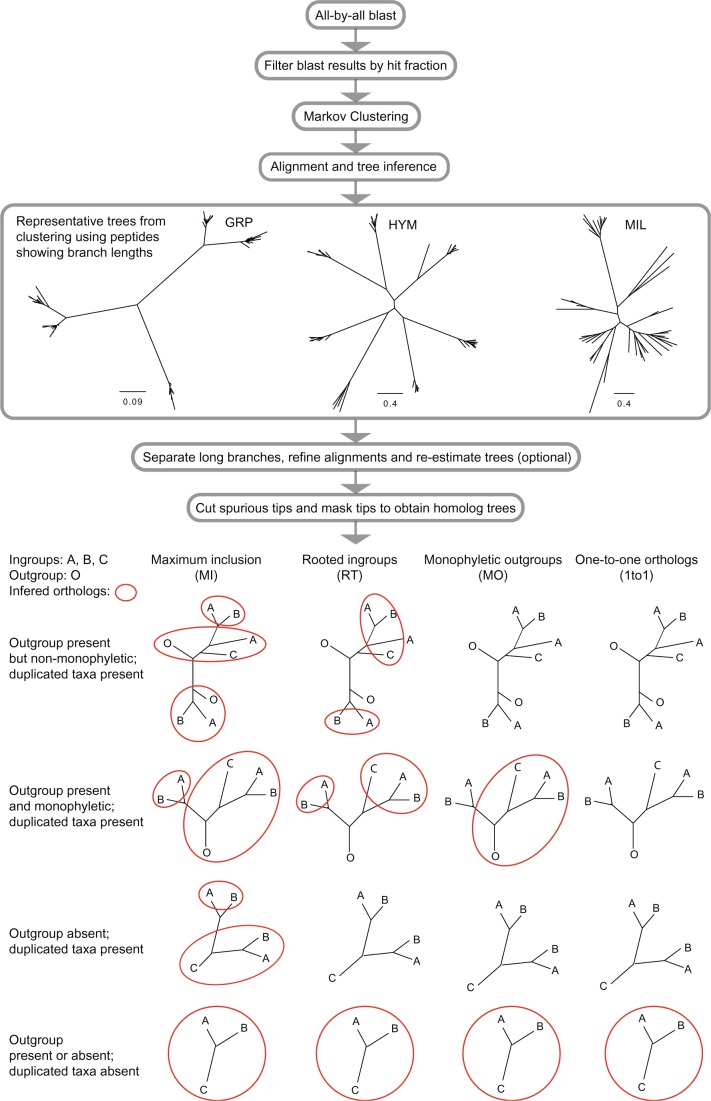
Flow chart of homology and orthology inferences. .

Our homology inference method starts with an all-by-all BLAST followed by clustering filtered BLAST hits using MCL (van Dongen 2000). For each cluster we align into a multiple sequence alignment, infer a phylogenetic tree using maximum likelihood, cut deep paralogs, and remove aberrant and redundant tips (fig. 1).

A number of methods can be used for conducting the initial homology search. For the analyses presented here, initial all-by-all homology searches were conducted using peptide sequences against a peptide database (BLASTP). In addition, we also conducted homology search using coding sequences (CDS) against a CDS database (BLASTN) in the GRP data set, which contains the most recently diverged group among the three we analyzed here. A third approach employed by Agalma (Dunn et al. 2013) uses transcripts translated by all six frames (TBLASTX) for homology search, and then simultaneously translate and align CDS using MACSE (Ranwez et al. 2011). This approach is promising for improving translation accuracy yet very time consuming and is currently limited to relatively small data sets.

Once a BLAST score has been calculated between each sequence pair, there are two general strategies for homology clustering. The simplest method is to filter out BLAST results that do not meet a minimal coverage percentage for either sequence (“hit fraction”; Chiu et al. 2006; Sanderson and McMahon 2007), then obtain clusters that are connected by the remaining hits. However, this approach is sensitive to both the value of the minimum hit fraction filter and large gene families that have sequences of intermediate completeness that can attract even less complete sequences. Our experience with using hit fraction alone is that it results in “snowballs” of gigantic clusters that are difficult to align because there is only partial overlap between many of the sequences. A second approach for homology clustering is to use a clustering algorithm, the most popular of which is MCL (van Dongen 2000). MCL is a general clustering algorithm that breaks any network of connected nodes into clusters by using the presence/absence of connections and the relative strength of those connections. It has the advantage of being extremely fast and efficient with computer memory. However, the algorithm uses only a single source of data (e.g., *E* values from BLAST hits) for clustering without taking the hierarchical structure of gene families into account. Because *E* values are dependent on data set size and sequence lengths (Altschul et al. 1990), the behavior and robustness of MCL have yet to be evaluated for data sets that include many partial sequences and sequence isoforms. In addition, because the *E* values produced by the BLAST algorithms frequently reach the lowest and most significant value (10^−^^180^) among large gene families, using MCL alone frequently produces clusters that are very large and difficult to align accurately. These large clusters are often removed from further analysis in existing pipelines, reducing the amount of usable data in these phylogenomic data sets.

To effectively separate gene families of various sizes, we developed a multistep procedure (fig. 1). Sequence similarity search results (here we use BLAST) are filtered using a minimum coverage fraction (hit fraction; here we use at least 0.4; Chiu et al. 2006; Sanderson and McMahon 2007) to remove hits from conserved motifs and short sequence fragments, and then clustered with MCL based on the filtered hits. The purpose of this initial clustering step is simply to form clusters of sizes that can be accurately aligned. Therefore values of the hit fraction cutoff and the inflation values in MCL are chosen to be as low as possible to ensure a coarse clustering that produces clusters containing less than a few thousand sequences each.

When a cluster contains deep duplications, the alignment will be poorly aligned, and the resulting phylogenetic tree will contain long branches subtending orthologs, especially in relatively recent data sets such as GRP and HYM (representative trees in fig. 1, showing trees from initial clusters using peptides). These branches often root orthologs at random internodes and interfere with orthology inference. One way to remove these deep duplications is to use all-by-all BLASTN using CDS instead of BLASTP using peptide sequences. This approach is effective only in recently diverged groups such as the grape family, and we found that with increasing divergence BLASTN is susceptible to Type II error. A second approach is using a higher inflation value in MCL (van Dongen 2000). By increasing the inflation value the clustering algorism is more sensitive to the contrasts in *E* values and connectivity among sequences, and tend to produce smaller clusters at the risk of breaking apart homologs at unexpected places. A third approach is to cut apart these deep duplications using a set of branch length cutoffs that are empirically determined by the distribution of branch lengths among ingroup taxa. In doing so, the accuracy of the alignments and the homolog trees are significantly improved. Although one can potentially detect subclusters that are significantly more distantly related among than within each subcluster, given the hierarchical structure of gene families, cutoffs for subclusters are often arbitrary and dependent on the phylogenetic distance among the ingroup taxa. Therefore here we simply set empirical branch length cutoffs to eliminate branches that are much older than diversification of orthologs. Finally, we trim spurious terminal branches that are much longer than sister branches that are usually a result of misassembly.

De novo assembled transcriptomes often have multiple isoforms for each gene that form monophyletic or paraphyletic tips on the gene tree. For phylogenomic purposes, only the isoform with the highest number of nonambiguous characters in the alignment is kept as the representative, with the rest removed. This procedure differs from Smith et al. (2011) and the “monophyly masking” step in Agalma (Dunn et al. 2013) in that instead of only masking monophyletic tip duplicates, we also mask paraphyletic grades of the same taxon, and we retain the isoform with the highest number of aligned characters after trimming instead of keeping a random one. Alternatively, one can keep the isoform with the shortest distance from its sister taxa or simply a random isoform. However, short branches often result from incomplete sequences, and a random isoform can contain poorly aligned sections from misassembly. By choosing the one with the most aligned characters after trimming we maximize the information retained. Another option is to pick either the longest isoform or the isoform with the highest read coverage from each isoform group (e.g., Trinity subcomponent). However, in practice, a subcomponent from an assembler (e.g., Trinity) does not always correspond to a gene and its splice variants (Grabherr et al. 2011). A previous benchmark study in a model plant species (Yang and Smith 2013) shows that chimeric transcripts exist in around 4% of Trinity assemblies, and picking the longest isoform alone will likely further increase the percentage of chimeric sequences. Picking the highest covered isoform per subcomponent, on the other hand, reduces the percentage of chimera from 4% to around 1% at the cost of reducing the total base pairs assembled by around 10% (Yang and Smith 2013). A final consideration for picking the representative isoform is alternative splicing. Splice variants with different exon content will introduce bias to distance-based orthology inference methods, whereas tree-based methods are less likely to be affected.

### Orthology Inference

We present four alternative orthology inference methods that may be used once homolog phylogenies have been inferred (fig. 1). The maximum inclusion (MI) method iteratively cuts out the subtree with the highest number of taxa without taxon duplication (Dunn et al. 2008, 2013; Smith et al. 2011). A second method iteratively searches for the subtree with the highest number of ingroup taxa, cuts it out as a rooted tree (RT) and infers gene duplications from root to tips. When duplicated taxa are found between the two sides at a bifurcating node, the side containing a smaller number of taxa is cut off. The third method looks for clusters with monophyletic outgroups (MO), roots the tree, and infers gene duplication in a similar way as RT. Both RT and MO are similar to the tree-pruning method implemented in TreeKO (Marcet-Houben and Gabaldón 2011) in that both traverse a rooted tree from root to tips and prune at nodes with taxon duplications. The two differ in that TreeKO considers all possible decompositions, calculates the pairwise distance between all candidate orthologs from two different homologs, and chooses one ortholog from each homolog that minimizes pair wise tree distance. However, given the incompleteness and noise in both transcriptome and low-coverage genome data, using a particular homolog as a reference for reconciliation bears the risk of introducing additional noise. Instead, we choose the decomposition that retains the highest number of taxa to maximize final matrix occupancy. Finally, we compare these results to only using homologs that had no duplicated taxon and are one-to-one (1to1) orthologs.

### Orthology Inferences from Example Data Sets

To demonstrate the utility of the methods presented here, we analyzed three data sets: GRP (Wen et al. 2013), MIL (Brewer and Bond 2013), and HYM (Johnson et al. 2013). The original authors provided peptides for HYM, whereas both MIL and GRP data were downloaded as raw reads from the NCBI Sequence Read Archive (SRA). For the MIL data set, our read filtering procedure differed from Brewer and Bond (2013). For details on deviations see Materials and Methods. Our quality filter removed 15–23% of read pairs (supplementary table S1, Supplementary Material online). Of the remaining read pairs, 0.03–0.82% contained adapters and were removed. The cleaned data sets contained 19–40 million read pairs each, 11–17% less than Brewer and Bond (2013). For the GRP data set, our quality filter removed 16–41% of read pairs (supplementary table S2, Supplementary Material online). Of the remaining read pairs 0.01–0.92% contained adaptors and were removed. After filtering 27–37 million read pairs for each taxon were used for de novo assembly.

**Fig. 2.**
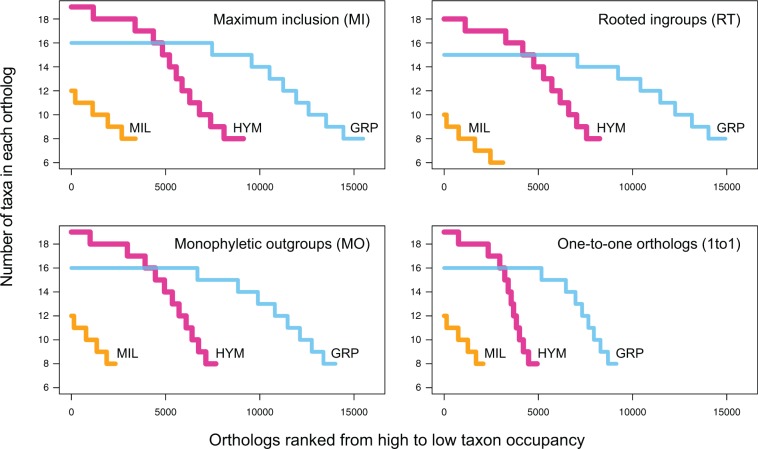
Ortholog taxon occupation ranked from high to low. Orthologs with less than eight taxa (six for MIL–RT) were not shown.

Homology and orthology inference were conducted using the methods as described above (for more details see Materials and Methods). The resulting ortholog occupancy curves were convex for HYM and GRP (fig. 2), indicating a high number of orthologs containing high percentage of taxa, whereas the almost straight curves for MIL indicate that relatively few orthologs have high percentage of taxa. The shapes were determined by the divergence time and the completeness of sequences in individual taxon (annotated genome vs. transcriptome/low-coverage genomes), whereas the orthology inference methods shifted the height and the slope of the curves.

#### The HYM Data Set

With seven out of 19 taxa from annotated genomes, the taxon occupancy curves of HYM were convex and all had a plateau that contained around 3,000–4,000 orthologs with complete or near complete taxon occupancy (fig. 2). One taxon in HYM, *Apterogyna* ZA01, was from a low-coverage genome and resulted in the narrow peak above the plateau in all four curves. Given that the outgroup *Nasonia vitripennis* had 12,925 genes, and the ingroup *Apis mellifera* had 10,570 genes, numbers of orthologs with at least eight taxa were high (MI: 9,128, RT: 8,251, MO: 7,665 and 1to1: 4,937).

Our RT method recovered 7,558 orthologs with at least nine taxa and 4,172 orthologs with at least 16 ingroup taxa, significantly more than the 5,214 and 3,018, respectively, from the Johnson et al. (2013) analyses using OrthologID (Chiu et al. 2006). Our final supermatrices contained orthologs with full taxon sets and at least 100 amino acids (aa) in trimmed alignments (MI: 1,160 loci, 620,150 aa; RT: 1,116 loci, 588,895 aa; MO: 992 loci, 525,061 aa; and 1to1: 761 loci, 369,371 aa), all with high amino acid occupancy (89.0%, 88.8%, 89.2% and 91.0%, respectively). Again these numbers are much higher than the 525 orthologs with full taxon occupancy recovered using OrthologID (Chiu et al. 2006; Johnson et al. 2013).

#### The GRP Data Set

With one annotated genomes and 15 transcriptomes, the GRP curves were also convex and all had a plateau that contained around 5,000–7,000 orthologs with full taxon occupancy (fig. 2). The large number of orthologs with full taxon occupancy likely reflects the fact that the GRP data set is relatively recent, with the split between the ingroups and the outgroup being approximately 95 Ma (Wen et al. 2013). Compared with the 29,971 genes in the *Vitis vinifera* (GRP) genome, approximately half of genes (a third for 1to1) had orthologs with at least eight taxa (MI: 15,488, RT: 14,929, MO: 14,016, and 1to1: 9,149).

We constructed the supermatrices using orthologs that had the full taxon set and containing at least 300 nt in trimmed alignments (MI: 7,462 loci, 10,925,506 nt; RT: 7,070 loci, 10,315,343 nt; MO: 6,686 loci, 9,870,949 nt; and 1to1: 5,166 loci, 7,403,388 nt). All four supermatrices had high nucleotide occupancy (89.6%, 89.1%, 89.2%, and 91.5%, respectively). Our ortholog sets with full taxon occupancy were much larger compared with the 417 (before filtering) and 229 (after filtering) orthologs by Wen et al. (2013) using Hcluster_sg (Li et al. 2006).

#### The MIL Data Set

With two annotated genomes and ten transcriptomes and ingroups dating back to more than 400 Ma (Brewer and Bond 2013), the MIL taxon occupancy curves were almost straight (fig. 2). The numbers of orthologs containing at least eight taxa were MI: 3,398, MO: 2,335, and 1to1: 2,075, whereas RT recovered 3,125 orthologs with at least six “ingroup” taxa (millipedes + *Lithobius*; see Materials and Methods). Among the four methods, MI recovered the highest number of orthologs, whereas the numbers of orhtologs recovered by both RT and MO were reduced by the high level of phylogenetic uncertainty among deep nodes. For the final supermatrices, we included orthologs that had no more than one taxon missing and each had at least 100 aa in the trimmed alignments (MI: 1,085, RT: 736, MO: 739, and 1to1: 712). Despite the variation in numbers, all four ortholog sets contained significantly more orthologs compared with the 221 orthologs recovered using HaMStR (Ebersberger et al. 2009) using similar alignment filtering procedures as Brewer and Bond (2013).

### Species Trees and Sources of Conflicts

We used concatenated supermatrices for species tree inference, partitioning by each locus. These supermatrices may contain conflicting phylogenetic signals due to hybridization, deep coalescence, contamination, and horizontal gene transfer. Noise and bias from assembly and orthology and tree inference may also complicate phylogenetic signal. To evaluate the presence of conflicting phylogenetic signal, we conducted serial jackknife analyses for each supermatrix keeping each locus intact.

#### The HYM Data Set

**Fig. 3.**
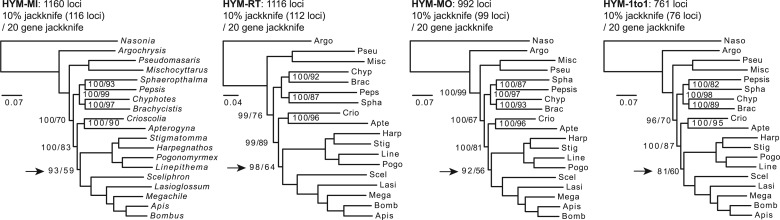
Maximum-likelihood analysis of the HYM data set. Taxon names were abbreviated to the first four letters of the genus names except the left-most tree. Orthology inference methods: MI, maximum inclusion; RT, extracting rooted ingroup clades; MO, monophyletic outgroups; 1to1, filtered one-to-one orthologs. All nodes received bootstrap and 30% jackknife support values of 100 and are not shown. Node labels are also not shown if all support values are 100. Arrows indicate nodes with relatively low support.

Species trees reconstructed from the HYM data set were overall highly consistent among all four orthology inference methods in topology, branch lengths, and support values (fig. 3). They had identical topologies to those in the analysis by Johnson et al. (2013). All branches received a support value of 100% from both the bootstrap and 30% jackknife analyses. Branches received less-than-perfect support values using STAR or PhyloNet in Johnson et al. (2013) similarly received less-than-perfect jackknife support values in our 10% and/or 20 gene jackknife analyses. The node uniting Formicidae and Apoidea (marked with an arrow in fig. 3) received 81–97% jackknife support with around 100 loci and around 60% with 20 loci. Given that five of the nine taxa in this clade were from annotated genomes and the entire tree was otherwise well supported, this Formicidae + Apoidea node warrants further investigation of the source of the conflict.

#### The GRP Data Set

**Fig. 4.**
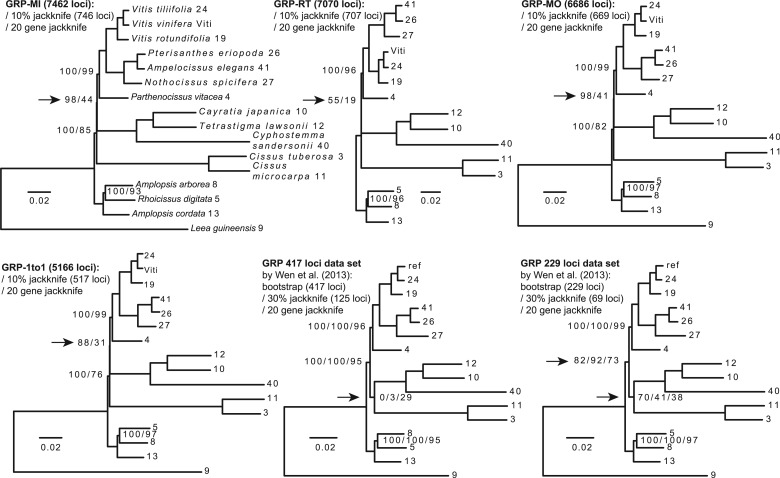
Maximum-likelihood analysis of the GRP CDS data set. Taxon names were replaced by the collection numbers except the top left tree. Orthology inference methods: MI, maximum inclusion; RT, extracting rooted ingroup clades; MO, monophyletic outgroups; 1to1, filtered one-to-one orthologs. Node labels are not shown when all support values are 100. Arrows indicate nodes with relatively low support.

The topology we recovered was well supported and in congruent with the topology recovered by Wen et al. (2013) except one of the basal nodes (fig. 4, indicated with arrows). We reanalyzed both the 417- and 229-gene CDS matrixes from Wen et al. (2013) with RAxML v7.3.5 (Stamatakis 2006), partitioning by gene. The two resulting trees similarly showed low support values at two of the basal nodes (fig. 4). The original species tree inference by Wen et al. (2013) did not apply any partition to the concatenated supermatrices and received bootstrap support of 100 for all nodes. Their topology stability test only included nodes among the ingroups without examining the uncertainty in the outgroup placement. Also, although their topological stability test involved serial subsampling by locus as we did here, they discarded the subsampled replicates when the maximum-likelihood tree had a topology different from the “standard topology.” They then calculated mean bootstrap values using only those replicates that agreed with the standard topology with no partitioning of subsampled matrixes. When partitioning was applied, all six supermatrices (fig. 4), four from our orthology inference, and two from Wen et al. (2013), showed strong conflicting signal among the deep nodes in Vitaceae. Therefore there is a need to take a closer look at the conflicting signals, the topology from plastid sequences, and perhaps also sequences from additional outgroup samples.

#### The MIL Data Set

**Fig. 5.**
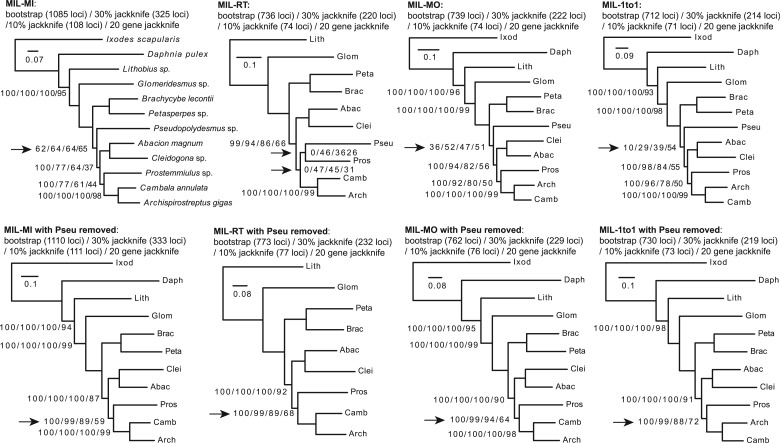
Maximum-likelihood analysis of the MIL. Taxon names were abbreviated to the first four letters of the genus names except the top left tree. Orthology inference methods: MI, maximum inclusion; RT, extracting rooted ingroup clades; MO, monophyletic outgroups; 1to1, filtered one-to-one orthologs. Node labels are not shown when all support values are 100. Arrows indicate nodes with relatively low support.

We recovered similar results to Brewer and Bond (2013) for the MIL data set. Despite the generally well-supported topology, the placement of *Pseudopolydesmus* was unstable. Clades including *Pseudopolydesmus* had low support values regardless of orthology inference methods used (fig. 5, arrows in upper four trees), and the support values in both RT and 1to1 decreased with increasing subsampling ratios. This indicates strong conflicting signals in the placement of *Pseudopolydesmus*. We subsequently removed *Pseudopolydesmus* from the initial RAxML output for homolog tree inference, trimmed tips, and carried out orthology and species tree inferences. By doing so the support values were significantly improved (fig. 5, lower four trees). Although the resulting species trees were well supported, the node uniting *Prostemmiulus*, *Cambala,* and *Archispirostreptus* (marked with an arrow in fig. 5, lower four trees) received support values of 88–94% when subsampling 10% of total genes and 59–72% when subsampling 20 genes. These values were relatively low compared with the rest of the tree and may deserve further investigations.

A number of other multilocus species tree methods have been used for reconstructing species trees and evaluating topological support using multiple genes without concatenation. Methods such as STAR (Liu, Yu, et al. 2009) and MP-EST (Liu et al. 2010) assume that coalescence is the only source of gene tree discordance, making bootstrap numbers derived from these models difficult to interpret. The program BUCKy does not assume a single source of discordance (Ané et al. 2007; Larget et al. 2010). However, BUCKy assumes that each individual gene has enough phylogenetic information and that the Markov chain Monte Carlo chains mixed well enough such that the posterior distribution of each gene tree reflects true phylogenetic uncertainty. For phylogenomic data sets with assembly error, partial and missing sequences, and genes with significant diversity in information content and molecular rate and therefore posterior distributions spread over many alternative topologies, BUCKy gives low concordance values across the tree that are difficult to interpret (Cui et al. 2013).

### Comparison among Methods of Homology and Orthology Inference

Among the four alternative orthology inference methods we examined, MI has the advantage of not requiring any outgroup information. It works well even in the absence of high-quality outgroups. However, in the presence of genome duplication, MI breaks orthologs each time duplicated taxon names are detected. Both RT and MO explicitly accommodate gene and genome duplications among the ingroups and are especially suitable for clades that have many gene/genome duplications. However, both require high-quality outgroup taxa that are phylogenetically distinct from the ingroup. In addition, RT requires outgroups that will not be included in the final ortholog sets and work best when there are multiple successive outgroups. The 1to1 strategy works for relatively small data sets, but otherwise is not likely to be useful. With transcriptome data sets that are both incomplete, redundant, and contain errors and isoforms, restricting to 1to1 relationships ignores the evolutionary history of gene families and is susceptible to repeated gene loss (De Smet et al. 2013). Finally, if the data set lacks high-quality outgroups and is complicated by genome duplications, the quality of orthology inference using any method will be problematic (table 1).

**Table 1.** Comparison of the Four Alternative Orthology Inference Methods Used in This Study.

High-Quality Outgroups	Genome Duplications	Examples	MI	RT	MO	1to1
Present	Absent	HYM, MIL	Good	Good	Good if only interested in low-copy genes	OK with a small number of taxa
Absent	Absent	Deep metazoan phylogeny; GRP	Good	Bad	Bad	OK with a small number of taxa
Present	Present	Many plant groups	Bad	Good	Good if only interested in low-copy genes	OK with a small number of taxa
Absent	Present	Many plant groups	Bad	Bad	Bad	May be the only choice

A final consideration for tree-based orthology inference is the computational cost. Our experience is that with increasing data set size, the computational bottleneck is at the stage of all-by-all homology search, which scales exponentially with the data set size. One possible modification is to infer a core homolog set using taxa with genome sequences, and then carry out homology search using sequences from the remaining taxa against these core homologs. This approach has the risk of missing novel genes that are not represented in the core homolog set. Once clusters are obtained, the alignment and tree inference steps can be easily distributed in many computer cores. With the recent advance in large-scale alignment and tree inference tools (Liu et al. 2012; Stamatakis et al. 2012; Katoh and Standley 2013), it is the time to fully take advantage of the information in gene trees to obtain more complete and more accurate homolog and ortholog sets.

## Conclusion

This study demonstrates the power of tree-based homology and orthology inference to recover significantly more usable data from short-read transcriptomic and low-coverage genomic data sets than existing heuristics. By reanalyzing three published data sets, we illustrate the procedures for obtaining cleaned and optimized homologs and orthologs, and show the utility of different strategies for resolving data sets of different age, completeness, rooting scenarios, and presence of genome duplications. We also illustrate the importance of including complete genomes, even if as members of the outgroup. The number of orthologs recovered can be dramatically improved with more complete data from individual taxa.

The real power of our tree-based procedure is that it preserves the full complement of evolutionary history present in each gene family. With this approach, future studies will be able to explore the rich information in these phylogenomic data sets such as functional and phylogenetic location of tree discordance, gene and genome duplications, shifts in molecular rates, and signatures of natural selection in nonmodel systems at an unprecedentedly broad scale.

## Materials and Methods

### Data Sets and Sequence Processing

For the MIL data set, nine transcriptomes from Brewer and Bond (2013) were downloaded from the SRA (accessions: SRX326775–SRX326777, SRX326779–SRX326784). Paired-end 50 bp reads were filtered using the read cleaning procedure from Yang and Smith (2013): Reads with average quality scores lower than 32 were removed; bases at the 3′-end with quality scores lower than 20 were trimmed, and only reads longer than 30 bp after trimming were kept. Both reads in a read pair were removed if one of the reads did not pass the quality filter. Adapter contamination was screened against the UniVec database (http://www.ncbi.nlm.nih.gov/tools/vecscreen/univec/, last accessed November 20, 2013) and, the Illumina TruSeq adapters and all vector containing read pairs were removed. This differed from the original publication in that we removed the entire read pair when an adapter was detected in either of the reads, instead of cutting off the first nine bases from all reads. Given the typical insertion size for Illumina RNA-seq libraries (∼130–200 bp), the presence of an adapter dimer (∼120 bp) would often render a read pair to be useless. All nine transcriptomes were assembled using Trinity version 20131110 with default settings (Grabherr et al. 2011), except that min_kmer_cov was set to 2 instead of the default value of 1, consistent with Brewer and Bond (2013). *Archispirostreptus gigas* EST sequences were downloaded from GenBank (4,008 in total, accessions FN194820–FN198827; Meusemann et al. 2010). All transcripts were translated using TransDecoder version 20131137 assisted by pfam domain information (Haas et al. 2013). Following Brewer and Bond (2013), additional proteome data of *Ixodes scapularis* were downloaded from VectorBase (www.vectorbase.org, last accessed November 19, 2013; Megy et al. 2012); and peptide sequences of *Daphnia pulex* were downloaded from the Joint Genome Institute http://genome.jgi-psf.org (filtered models v1.1, last accessed November 19, 2013; Colbourne et al. 2011).

We suggest that future NCBI SRA submissions contain information about what kit and modifications were used for library preparation, the adapters used and the distribution of insertion sizes in either or both the SRA submission and the methods narratives, even when the library preparation was outsourced. Such information would greatly facilitate effective reuse of these archived data sets.

For the GRP data set, all 15 transcriptomes generated by Wen et al. (2013) were downloaded from GenBank (SRA accessions SRX286217–SRX286231). Paired-end 90 bp reads were filtered by quality scores, and adaptor contamination was removed with the same procedure as for MIL. The remaining reads were assembled using Trinity version 20140413 with default settings (Grabherr et al. 2011), and translated using TransDecoder version rel16JAN2014 assisted by pfam domain information (Haas et al. 2013). CDS of *V*. *vinifera* were downloaded from the Phytozome database v9.1 (Jaillon et al. 2007; Goodstein et al. 2012).

For the HYM data set, all peptide sequences were kindly provided by the authors (Johnson et al. 2013), including peptide sequences from additional studies (http://www.ncbi.nlm.nih.gov/bioproject/66515; https://www.hgsc.bcm.edu/arthropods/bumble-bee-genome-project; Weinstock et al. 2006; Bonasio et al. 2010; Werren et al. 2010; Smith, Smith, et al. 2011; Smith, Zimin, et al. 2011; Kocher et al. 2013). All peptides were reduced with cd-hit (-c 0.99 -n 5), and CDSwere reduced with cd-hit-est (-c 0.995 -n 10 -r 1; Fu et al. 2012).

### Homology Inference

Homology searches were carried out using all-by-all BLASTP from peptides of all three data sets and an additional all-by-all BLASTN search using CDS from GRP. All BLAST searches used an *E* value cutoff of 1 and max_target_seqs set to 100. BLAST output was filtered by a requirement that the hit fraction being at least 0.4 (Chiu et al. 2006; Sanderson and McMahon 2007). MCL (MCL v12-068; van Dongen 2000; Enright et al. 2002; van Dongen and Abreu-Goodger 2012) was performed on filtered all-by-all BLASTP hits, with the *E* value cutoff set to 10^−^^5^ and an inflation value of 1.4. Ends with no BLAST hits, presumably from misassembly and/or frameshift, were cut off. Remaining sequences shorter than 40 characters were removed, and clusters smaller than eight taxa were removed. Each resulting cluster was aligned using MAFFT v7.043b (–genafpair–maxiterate 1000 if less than 1,000 sequences; –auto when 1,000 or more sequences; Katoh and Standley 2013).

Clusters may include divergent sequences and the alignments therefore require refinement (the optional step; fig. 1). Alignments that included 200 or more sequences were refined with SATé v2.2.7 (Liu, Raghavan, et al. 2009; Liu et al. 2012) starting with alignments from MAFFT. Alignments were trimmed with Phyutility v2.2.6 (-clean 0.01) and an initial phylogenetic tree estimated with FastTree v. 2.1.7 (Price et al. 2010). The resulting trees often contain misassembly, recombination, or paralogs with deep splits that formed long branches. These long branches (1.5 for MIL, 1.2 for HYM, and 0.6 for GRP with peptides) were cut and sequences from each subtree were realigned using MAFFT followed by SATé as the previous step. As for the GRP CDS data set the initial alignments using MAFFT were well aligned and were directly used in subsequent steps.

Resulting alignments were trimmed with Phyutility (-clean 0.1). Maximum-likelihood phylogenies were inferred using RAxML v7.3.5 (Stamatakis 2006) with the model PROTCATWAG for peptides and the model GTRCAT for CDS. The resulting trees occasionally still had unusually long tips that likely arose from misassembly and/or frameshift. A tip was removed if it was more than 10 times longer than the average distance to tips seen in its sister clade, and was longer than 0.75 for MIL, 0.6 for HYM, or 0.1 for GRP. When monophyletic or paraphyletic tips from the same taxa were present in a tree, only the one with the highest number of nonambiguous characters in the trimmed alignment was kept as the representative, with the rest removed (Dunn et al. 2008; Smith, Wilson, et al. 2011).

Finally, branches longer than 1.5 for MIL, 1.0 for HYM, and 0.3 for GRP were cut. The resulting trees were called homologous gene trees. Three homologous gene tree sets were obtained: MIL, GRP (CDS only onwards), and HYM.

### Orthology Inference

All homologous gene trees were further pruned to produce the orthologs containing one sequence per species. Four alternative strategies were applied for each homolog set (fig. 1): MI, RT, MO, and 1to1.

We used the MI method (Dunn et al. 2008, 2013; Smith, Wilson, et al. 2011) to search the homologous gene tree for the subtree that had the highest number of taxa without any taxon repeat and cut it off as an ortholog (fig. 1). We then continued searching the remaining tree with the same cutting criteria until no subtree with at least eight nonrepeating taxa could be found. As the remaining tree occasionally contained tips subtended by long branches as a result of leftover from pruning off orthologs, a tip was removed if it was more than ten times longer than the average distance to tips seen in its sister clade, and was longer than 0.4 for MIL, 0.3 for HYM, or 0.1 for GRP.

The RT (fig. 1) strategy uses predefined outgroups to orient and extract ingroup clades, and then infer locations of gene duplication events from the extracted ingroup clade. We first iteratively searched for the subtree that had the highest number of ingroup taxa regardless of taxon duplications and cut it off as a rooted tree. We then traversed this rooted ingroup tree from the root toward the tips, while inferring locations of gene duplication events from deep ones to more recent ones by looking for taxon duplication between the two sides at each bifurcation. When a gene duplication was found at a node, the side with a smaller number of taxa was pruned to maximize taxon occupancy in the remaining tree. This paralog pruning procedure was carried out iteratively on all subtrees until no taxon duplication was left in any subtree with at least eight taxa. As for homologs that lack outgroups, only those with no taxon duplication were included as unrooted ortholog trees. Homologs with duplicated taxa but no outgroup taxa were ignored due to difficulties in inferring locations of gene duplications without rooting. The MIL data set included three non-millipede arthropods forming a successive grade to the millipedes. Two of the more distantly related outgroups, *Ixodes,* and *Daphnia* were both from genome annotations; whereas the third taxa, *Lithobius*, was the closest to the millipedes and was sampled using RNA-seq. Therefore, we regarded both *Ixodes* and *Daphnia* as “outgroup” in our outgroup-based orthology inferences, and treated *Lithobius* as a member of the ingroup. As for HYM, one single nonaculeate genome-derived *Nasonia* was used as the outgroup following the original analysis. For GRP, the transcriptome-derived outgroup *Leea* was used following the original analysis.

Although RT is effective for data sets with genome duplications, outgroups used for extracting rooted clades will be absent from the final orthologs. A modification for RT is to root ingroups only when the outgroups are monophyletic and nonrepeating (MO). By doing so, all taxa will be preserved in the resulting ortholog sets, while losing a fraction of homologs.

Finally, we also present results using only homologs without any taxon repeat (1to1) for completeness, similar to procedures that use reciprocal criteria (Ebersberger et al. 2009; Schreiber et al. 2009).

### Estimating Species Tree

Following ortholog inference, an alignment was obtained for each ortholog by extracting aligned sequences from the homologs. The resulting alignments were trimmed with Phyutility (-clean 0.3) for HYM and GRP. Because MIL had more divergent sequences, alignments were trimmed with Gblocks v0.91b (Talavera and Castresana 2007; Soria-Carrasco and Castresana 2008) using the same settings as of Brewer and Bond (2013). For the final supermatrices, we only included trimmed ortholog alignments that were at least 100 aa for MIL and HYM, or 300 nt for GRP in trimmed length, and each ortholog had no more than one missing taxon for MIL, and only orthologs with the full taxon set for HYM and GRP.

Maximum-likelihood trees were estimated using RAxML with PROTCATWAG model for the MIL and HYM data sets, and in ExaML v2.0.4 (Stamatakis et al. 2012) with GTRCAT for the GRP data set, partitioning each ortholog. Conflicts among orthologs were estimated by 200 jackknife replicates each resampling a fixed proportion (10% or 30%), or a fixed number of 20 orthologs keeping each ortholog intact. Finally, despite the recognition that the bootstrap method may provide a poor measurement of confidence in genome-wide data sets (Salichos and Rokas 2013), we carried out 200 rapid bootstrap replicates for the HYM and MIL data sets to compare to their respective original analyses.

## Supplementary Material

Supplementary tables S1 and S2 are available at *Molecular Biology and Evolution* online (http://www.mbe.oxfordjournals.org/).

Supplementary Data
