# Supplementary material for: Orthology Inference in Nonmodel Organisms Using Transcriptomes and Low-Coverage Genomes: Improving Accuracy and Matrix Occupancy for Phylogenomics
Source: Mol Biol Evol. 2014 Aug 25;31(11):3081–92. doi: 10.1093/molbev/msu245 (PMC4209138; doi:10.1093/molbev/msu245)
Supplement: Supplementary Data [file supp_31_11_3081__index.html]

Orthology inference in non-model organisms using transcriptomes and low-coverage genomes: improving accuracy and matrix occupancy for phylogenomics — Orthology Inference in Nonmodel Organisms Using Transcriptomes and Low-Coverage Genomes: Improving Accuracy and Matrix Occupancy for Phylogenomics — Orthology Inference in Nonmodel Organisms Using Transcriptomes and Low-Coverage Genomes: Improving Accuracy and Matrix Occupancy for Phylogenomics — Supplementary Data 

# Orthology Inference in Nonmodel Organisms Using Transcriptomes and Low-Coverage Genomes: Improving Accuracy and Matrix Occupancy for Phylogenomics

## Supplementary Data

files

**Files in this Data Supplement:**

- Supplementary Data - xlsx file
- Supplementary Data - xlsx file
